# Aspirin sensitizes osimertinib‐resistant NSCLC cells *in vitro* and *in vivo* via Bim‐dependent apoptosis induction

**DOI:** 10.1002/1878-0261.12682

**Published:** 2020-05-05

**Authors:** Rui Han, Shuai Hao, Conghua Lu, Chong Zhang, Caiyu Lin, Li Li, Yubo Wang, Chen Hu, Yong He

**Affiliations:** ^1^ Department of Respiratory Disease Daping Hospital Army Medical University Chongqing China; ^2^ Department of Ultrasound The First Affiliated Hospital of Chongqing Medical University China

**Keywords:** apoptosis, aspirin, EGFR‐TKI, osimertinib, resistance

## Abstract

Osimertinib, a third‐generation irreversible epidermal growth factor receptor tyrosine kinase inhibitor (EGFR‐TKI), provides marked clinical benefit for patients with EGFR‐activating mutations. Unfortunately, limited treatments exist for patients who acquire osimertinib resistance. We observed two ‘special’ patients who regained an antitumor response with osimertinib plus aspirin treatment. As previous data indicate that aspirin induces antiproliferative effects in tumor cells, we designed a preclinical study to explore whether aspirin combined with osimertinib could synergistically sensitize osimertinib‐resistant non‐small‐cell lung cancer (NSCLC) cells. The effects of combined treatment with osimertinib and aspirin on osimertinib‐resistant NSCLC cell lines were examined *in vitro* and *in vivo*. The combination of osimertinib and aspirin induced strong antiproliferative and proapoptotic effects in osimertinib‐resistant NSCLC cells through inhibition of Akt/FoxO3a signaling component phosphorylation and increased Bim expression. Furthermore, Bim knockdown by siRNA significantly attenuated osimertinib resensitization by aspirin. *In vivo*, combination of aspirin and osimertinib significantly decreased tumor growth of PC‐9GROR cell xenografts. Data of patients with NSCLC who received osimertinib treatment at Daping Hospital between January 2015 and January 2019 were reviewed retrospectively. According to clinical data for 45 patients with NSCLC, retrospective analysis showed that the median progression‐free survival was significantly longer in the osimertinib plus aspirin group than in the osimertinib group. In summary, aspirin synergistically enhances the antitumor activity of osimertinib in osimertinib‐resistant lung cancer cells through promoting Bim‐dependent apoptosis. This combination therapy may be effective in overcoming acquired resistance to osimertinib and prolonging survival in patients with NSCLC.

## Graphical abstract

Aspirin synergistically enhances the antitumor activity of osimertinib in osimertinib‐resistant lung cancer cells through promoting Bim‐dependent apoptosis. This combination therapy may be effective in overcoming acquired resistance to osimertinib and prolonging survival in patients with non‐small‐cell lung cancer.

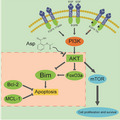

**Abbreviations**
EGFR‐TKI: epidermal growth factor receptor tyrosine kinase inhibitor
IGF: insulin‐like growth factor
NSAIDs: nonsteroidal anti‐inflammatory drugs
NSCLC: non‐small‐cell lung cancer
PFS: progression‐free survival
PR: partial response
siRNA: small‐interfering RNA

## 1 Introduction

Lung cancer is the leading cause of cancer‐related death globally (Bray *et al.*, 2018). Epidermal growth factor receptor tyrosine kinase inhibitors (EGFR‐TKIs) offer significant clinical benefit for patients with EGFR mutations (Fukuoka *et al.*, 2011; Zhou *et al.*, 2011). Osimertinib, a third‐generation EGFR‐TKI, is currently the standard treatment for patients with progression after first‐ or second‐generation EGFR‐TKIs because of the T790M mutation or as a first‐line treatment for EGFR mutation‐positive advanced non‐small‐cell lung cancer (NSCLC) (Mok *et al.*, 2017; Ramalingam *et al.*, 2018; Soria *et al.*, 2018). Unfortunately, acquired resistance to osimertinib is inevitable (Yang *et al.*, 2018). Thus, innovative treatment strategies are urgently needed to overcome acquired resistance to osimertinib.

Although osimertinib resistance is not fully elucidated, the mechanisms have been divided into EGFR‐dependent mechanisms such as EGFR mutations (C797S and L798I) and EGFR‐independent mechanisms such as bypass of MET or ErbB2 signaling activation (Oxnard *et al.*, 2018; Yang *et al.*, 2018). Various mechanisms of osimertinib resistance contribute to the difficulty in finding ways to overcome it, and there are no effective strategies currently. Therefore, it is of great importance to investigate new resistance mechanisms as well as strategies to cope with resistance.

Recent studies have shown that the therapeutic efficacy of osimertinib is closely related to the induction of apoptosis. Research involving two osimertinib‐resistant cell lines strongly suggests that the critical mechanism by which sensitive EGFR‐mutant NSCLC cells become resistant to osimertinib‐mediated apoptosis induction occurs via an inability of osimertinib to modulate B‐cell lymphoma 2‐like 11 (Bim) and myeloid cell leukemia sequence 1 (Mcl‐1) levels (Shi *et al.*, 2017). Thus, promoting apoptosis may overcome osimertinib resistance in osimertinib‐resistant cell lines, and identifying a clinically available drug that induces apoptosis may be a feasible strategy for overcoming osimertinib resistance.

Acetylsalicylic acid (aspirin), one of the most common nonsteroidal anti‐inflammatory drugs (NSAIDs), is widely used as an antiplatelet agent to prevent myocardial infarction and stroke. Accumulated recent studies have reported the correlation with long‐term aspirin application and low risk of cancer mortality (Bradley *et al.*, 2016; Lucotti *et al.*, 2019; Merritt *et al.*, 2018; Simon *et al.*, 2018). What's more, aspirin has been recommended for primary prevention of colorectal cancer (Brenner and Chen, 2018) and can also induce apoptosis in tumor cells, as demonstrated by its ability to act synergistically with erlotinib to induce apoptosis in human EGFR‐mutant NSCLC cell lines (Hu *et al.*, 2018). Consequently, we postulated aspirin might overcome osimertinib resistance by inducing apoptosis.

In our study, we evaluated whether aspirin in combination with osimertinib has synergistic antitumor activity using various *in vitro* and *in vivo* approaches, including the thiazolyl blue tetrazolium bromide (MTT) assay, flow cytometry, western blot assay, and xenografts. Our investigations showed aspirin can sensitize osimertinib resistance NSCLC cells to osimertinib *in vitro* and *in vivo* by inducing apoptosis, which is dependent on inhibition of Akt/FoxO3a signaling component phosphorylation and increased Bim expression. We thereby provide rationale and evidence for considering the use of aspirin in combination with osimertinib to overcome osimertinib resistance in NSCLC patients.

## 2 Materials and methods

### 2.1 Cell lines and reagents

Gefitinib‐resistant PC‐9GR cells were donated by J. Xu and M. Liu from Guangzhou Medical University (China). These cells harbored EGFR 19 Del and T790M mutations and were sensitive to osimertinib. Erlotinib‐resistant H1650‐M3 cells were kindly provided by R. Sordella. H1975 cells were obtained from American Type Culture Collection, and these cells harbored EGFR L858R and T790M mutations and were sensitive to osimertinib. All the osimertinib‐resistant PC‐9GROR, H1975‐OR cell lines and rociletinib (CO1686)‐resistant PC‐9GRCOR, H1975‐COR cell lines were constructed in our laboratory. The corresponding osimertinib parental and resistant cells were first treated with osimertinib at the concentration of IC50 for 2 weeks and then were treated with a higher concentration for another 3 weeks sufficient to kill nearly all the parental cells. Finally, the remaining resistant clones were seeded into single cell per well and were cultured continuously in the presence of osimertinib (Li *et al.*, 2019). Rociletinib (CO1686)‐resistant cell lines were established in a manner similar to the osimertinib‐resistant cell lines (Pan *et al.*, 2018). Treatment with 200 µmol·L^−1^ aspirin alone for 48 h slightly decreased the viability of PC‐9GR, H1975, H1650‐M3, PC‐9GROR, H1975‐OR, PC‐9GRCOR, and H1975‐COR cells (Fig. S1). Therefore, we chose a combination of 200 µmol·L^−1^ aspirin and different doses of osimertinib to treat cell lines resistant to third‐generation EGFR‐TKIs. All the cells were cultured in Roswell Park Memorial Institute (RPMI)‐1640 (HyClone, Logan, UT, USA) medium supplemented with 10% fetal bovine serum (Gibco, Waltham, MA, USA), 100 U·mL^−1^ penicillin (HyClone), and 100 µg·mL^−1^ streptomycin (HyClone) at 37 °C in 5% CO_2_ and 90% humidity.

Osimertinib (Tagrisso) was obtained from Astra Zeneca (London, UK), rociletinib was purchased from Selleck Chemicals (Houston, TX, USA), gefitinib (Iressa) was purchased from MedChemExpress (Monmouth Junction, NJ, USA), erlotinib (Tarceva) was obtained from Cayman Chemical (Ann Arbor, MI, USA), aspirin (A2093) was purchased from Sigma‐Aldrich (Darmstadt, Germany), and aspirin (S3017) was obtained from Selleck Chemicals. Osimertinib, rociletinib, gefitinib, erlotinib, and aspirin were dissolved in DMSO. Human insulin‐like growth factor (IGF)‐1 (#100‐11) was purchased from PeproTech (Ribeirão Preto‐SP, Brazil). Antibodies against Bim (#2933S), Mcl‐1 (#5453S), p‐Mcl‐1 (#14765S), B‐cell lymphoma 2 (Bcl‐2; #2870S), Bcl‐2‐associated X protein (BAX; #5023S), Akt (#9272S), phospho(Ser473)‐Akt (#4060S), FoxO3a (#2497S), phospho‐FoxO3a (#9466S), and glyceraldehyde‐3‐phosphate dehydrogenase (GAPDH; #2118S) were obtained from Cell Signaling Technology (Danvers, MA, USA).

### 2.2 Cell proliferation assays

Cell viability was assessed using MTT assays (Takahashi *et al.*, 2018). Cells were seeded at 2000 cells per well in 96‐well plates and incubated in RPMI‐1640 + 10% FBS. MTT assay for parental PC‐9GR, H1975 cells and their corresponding osimertinib‐ or rociletinib‐resistant cells treated with DMSO indicated doses of osimertinib (1 μmol·L^−1^ in PC‐9GROR cells, 3 μmol·L^−1^ in H1975‐OR cells), osimertinib plus aspirin (200 μmol·L^−1^) or rociletinib (0.5 μmol·L^−1^ in PC‐9GROR cells, 3 μmol·L^−1^ in H1975‐OR cells), and rociletinib plus aspirin for 48 h. Experiments were performed in triplicate, and data are mean ± SEM.

### 2.3 Apoptosis assessment

Apoptosis was detected using flow cytometry analysis and the terminal deoxynucleotidyl transferase dUTP nick end labeling (TUNEL) staining assay. Cells were collected by trypsinization at 48 h after different treatments, washed three times with PBS, and resuspended at a density of 1 × 10^6^ cells·mL^−1^. After double staining with PI for 30 min at ambient temperature in the dark using FITC Annexin V Apoptosis Detection Kit I, the cells were analyzed using a flow cytometer (Beckman Coulter Navios, Brea, CA, USA).

### 2.4 Reverse transcription–polymerase chain reaction

Total cellular RNA was extracted from the cells using TRIzol reagent (#15596026; Thermo Scientific, Waltham, MA, USA) and reverse‐transcribed using an iScript gDNA clear cDNA Synthesis Kit (Bio‐Rad, Hercules, CA, USA). The mRNA expression was amplified by 5× Pfu PCR MasterMix using a CFX96 Touch System (Bio‐Rad). The forward and reverse primers for human Bim were 5′‐CCACCAATGGAAAAGGTTCA‐3′ and 5′‐GGCACAGCCTCTATGGAGAA‐3′, respectively, and the forward and reverse primers for human GAPDH were 5′‐GGTGAAGGTCGGAGTCAACG‐3′ and 5′‐CAAAGTTGTCATGGATGACC‐3′, respectively. Amplified fragments in DNA loading buffer were subjected to agarose gel electrophoresis for 40 min at 110 V, followed by labeling with GoldView staining solution and visualization using the ChemiDoc Touch System (Bio‐Rad).

### 2.5 Western blot analysis

Cells were scraped from culture vessels and washed twice with PBS. The cells were then lysed for 30 min at 4 °C in radio‐immunoprecipitation assay buffer (Sigma‐Aldrich). The cellular debris was removed by centrifugation at 12 000 ***g*** for 30 min at 4 °C, and the protein concentration was determined using the Bradford method (Millipore, Darmstadt, Germany). Equal amounts of protein were subjected to gel electrophoresis for 2 h at 110 V, followed with which were transferred into polyvinylidene difluoride membranes (90 min, 200 mA) (Millipore). Then, the membranes were blocked with 5% bovine serum albumin for 1 h at room temperature and incubated overnight at 4 °C with primary antibodies. Subsequently, the membranes were washed and incubated with 0.02 µg·mL^−1^ horseradish peroxidase‐conjugated goat anti‐rabbit (Cell Signaling Technology) for 1 h, followed by visualization with ChemiDoc Touch System (Bio‐Rad).

### 2.6 Xenograft studies

All animal protocols were approved by the Ethics Committee of Army Medical University. Four‐week‐old female BALB/c A‐nu mice (Laboratory Animal Center of Army Medical University, Chongqing, China) were injected subcutaneously into the back (next to the left forelimb) with 2 × 10^6^ PC‐9GROR cells. Once the tumors reached a size of approximately 50 mm^3^ (within 5–7 days), the mice were randomly assigned to one of four groups (5 mice/group). Based on other prior studies, the mice were given osimertinib (5 mg/kg), aspirin (20 mg·kg^−1^), and a combination of osimertinib and aspirin by means of intragastric administration (Chen *et al.*, 2018; Shi *et al.*, 2017). The tumor volume was calculated as (length × width^2^)/2 and measured twice per week. The mice were maintained in individual ventilated cages in compliance with institutional guidelines. They were monitored for 5 weeks until euthanasia. For western blot analysis, the tumors were homogenized in protein lysis buffer. To assess survival, once the tumors reached a size of approximately 50 mm^3^, the animals were randomly assigned to one of three groups (10 mice/group) and treated with intragastric administration once daily with water alone or water containing osimertinib (5 mg·kg^−1^) or a combination of osimertinib and aspirin. The mice were monitored for 100 days until they were euthanized.

### 2.7 Patient information

This study was approved by the Institutional Review Board of Daping Hospital, Army Military Medical University. All patients provided informed consent for this study and gave permission to the entire study. Disease progression was confirmed in each patient according to the RECIST 1.1 criteria. Clinical information was retrospectively obtained from 45 patients presenting with NSCLC who had received first‐generation EGFR‐TKI (gefitinib or erlotinib) therapy and were resistant due to T790M mutation. Then, the patients had received osimertinib therapy in Daping Hospital between January 2015 and January 2019. These patients were histologically or cytologically confirmed to harbor either exon 19 deletion or L858R mutation and T790M mutation. All participants provided written consent before enrollment. This study was approved by the Ethics Committees of the Daping Hospital, Army Medical University, and conformed to the tenets of the Declaration of Helsinki.

### 2.8 Statistical analysis

All data were analyzed using Student's *t*‐test, and the results are expressed as the mean ± SEM of triplicate samples. Survival percentages over time were estimated using the Kaplan–Meier method. Statistical significance was assumed at an alpha value of *P* < 0.05.

## 3 Results

### 3.1 Aspirin sensitizes osimertinib‐resistant NSCLC patients to osimertinib

**Fig. 1:**
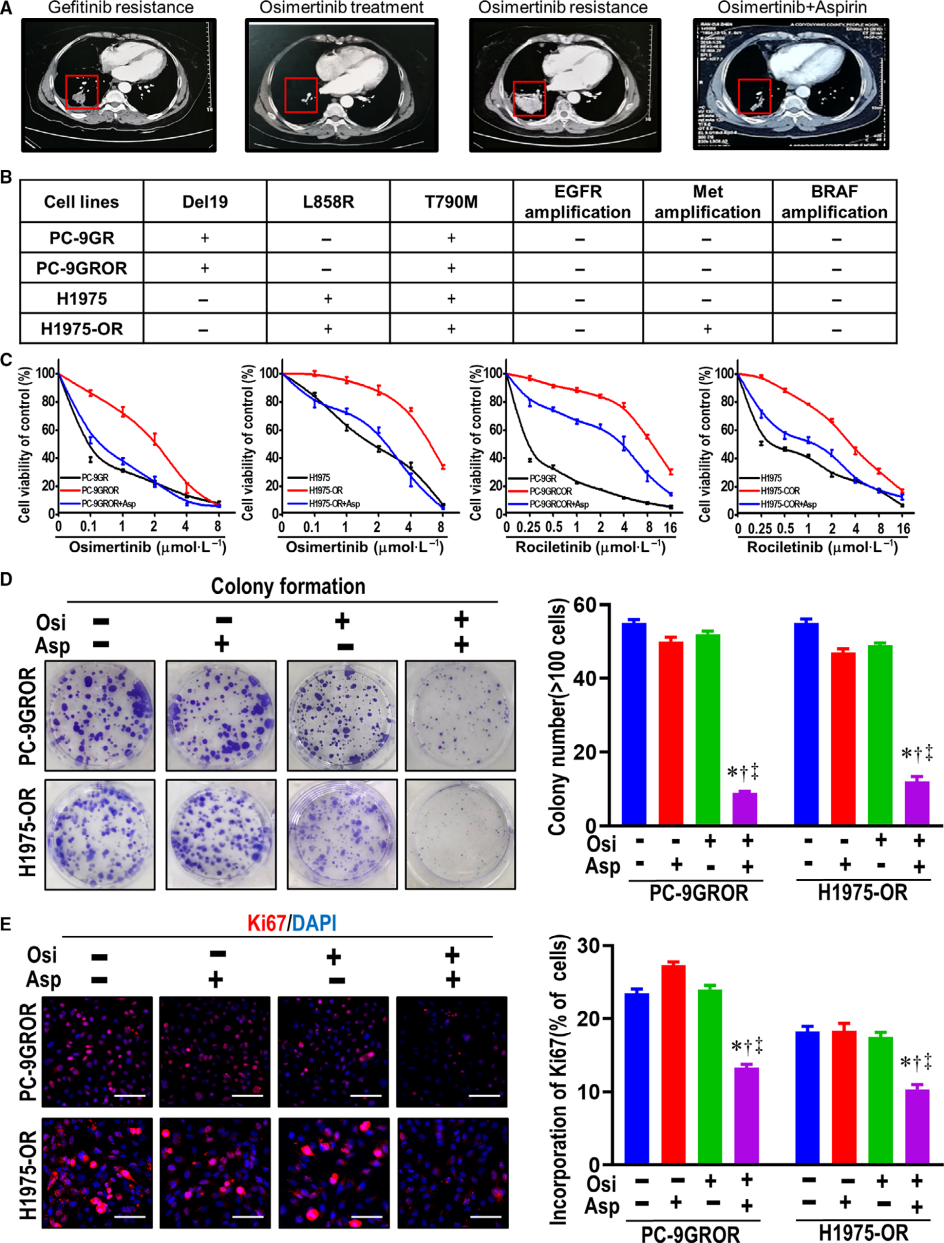
Aspirin sensitizes osimertinib in osimertinib‐resistant NSCLC patients and osimertinib‐resistant cells. (A) The chest CT examination showed the size of the patient's tumor reduction followed with aspirin and osimertinib concurrent treatment. (B) Summary of the gene alterations in each parental and resistant cell lines detected by next‐generation sequencing. (C) Cell viability was analyzed by MTT assay in parental PC‐9GR, H1975 cells and their corresponding osimertinib‐ and rociletinib‐resistant cells. Mean ± SEM. (D) Representative images of showing the colony formation ability of resistant PC‐9GROR and H1975‐OR cells. Histogram showed the colony cell numbers in the indicated groups. The colony formation rates are shown as the mean ± SD from three independent experiments. *P < 0.05 compared with control; ^†^ *P* < 0.05 compared with aspirin treatment alone; ^‡^ *P* < 0.01 compared with osimertinib treatment alone. (E) Ki67 proliferation assay of resistant PC‐9GROR cells and H1975‐OR cells with various treatments. Histogram showed the incorporation cells in the indicated groups, respectively Scale bars: 100 μm. Data are from three independent assays and are shown as the mean ± SD. *P < 0.05 compared with control; ^†^ *P* < 0.05 compared with aspirin treatment alone; ^‡^ *P* < 0.01 compared with osimertinib treatment alone. DAPI, 4′,6‐diamidino‐2‐phenylindole; Asp, aspirin; Osi, osimertinib.

We incidentally observed osimertinib sensitization in two patients with advanced NSCLC. The two *EGFR*‐positive advanced lung adenocarcinoma never smoker female patients (58 years old, T4N2M1, L858R; 61 years old, T2N3M1, 19Del) were administered gefitinib and subsequent osimertinib. Both patients were received 80 mg osimertinib once daily and achieved partial response (PR), with a progression‐free survival (PFS) of 26.5 or 28 months, respectively. Due to the retention of EGFR T790M and no other resistance mechanism was detected at disease progression, these patients remained on osimertinib. In addition, they both experienced cardiovascular disease, and 100 mg aspirin daily was coadministered with osimertinib. The addition of aspirin effectively reversed osimertinib resistance and resulted in a significant reduction in the size of the primary lesion, achieving the best response of PR, with a PFS of 7 months (Fig. 1A and Fig. S2). The two cases prompt that aspirin and osimertinib may have synergistic antitumor activity.

### 3.2 Aspirin sensitizes osimertinib in osimertinib‐resistant NSCLC cell lines

To detect the effects of aspirin *in vitro*, we used the osimertinib‐resistant NSCLC PC‐9GROR and H1975‐OR cells, which are highly resistant to osimertinib (Li *et al.*, 2019). Next‐generation sequencing showed the presence of an EGFR‐sensitive mutation (Del19 or L858R) and the T790M mutation in both NSCLC cell lines; however, MET amplification was detected only in the H1975‐OR cell line (Fig. 1B). The MTT assay showed that PC‐9GR and H1975 cells are sensitive to osimertinib, whereas that PC‐9GROR and H1975‐OR cells are highly resistant to osimertinib. Treatment with 200 µmol·L^−1^ aspirin resensitized PC‐9GROR cells and H1975‐OR cells to osimertinib (Fig. 1C and Fig. S3A). Moreover, treatment with 200 µmol·L^−1^ aspirin resensitized PC‐9GRCOR and H1975‐COR cells to the other third‐generation EGFR‐TKI rociletinib (Fig. 1C and Fig. S3B). We also found that aspirin was able to resensitize the first‐generation EGFR‐TKIs gefitinib and erlotinib (Fig. S3C,D). The results from both colony formation and Ki67 assays confirmed enhanced inhibitory effects of aspirin plus osimertinib in PC‐9GROR and H1975‐OR cells (Fig. 1D,E). Taken together, these results indicate that aspirin can resensitize osimertinib to osimertinib‐resistant NSCLC cell lines.

### 3.3 Reduced apoptosis leads to osimertinib resistance in EGFR‐mutant NSCLC cell lines

**Fig. 2:**
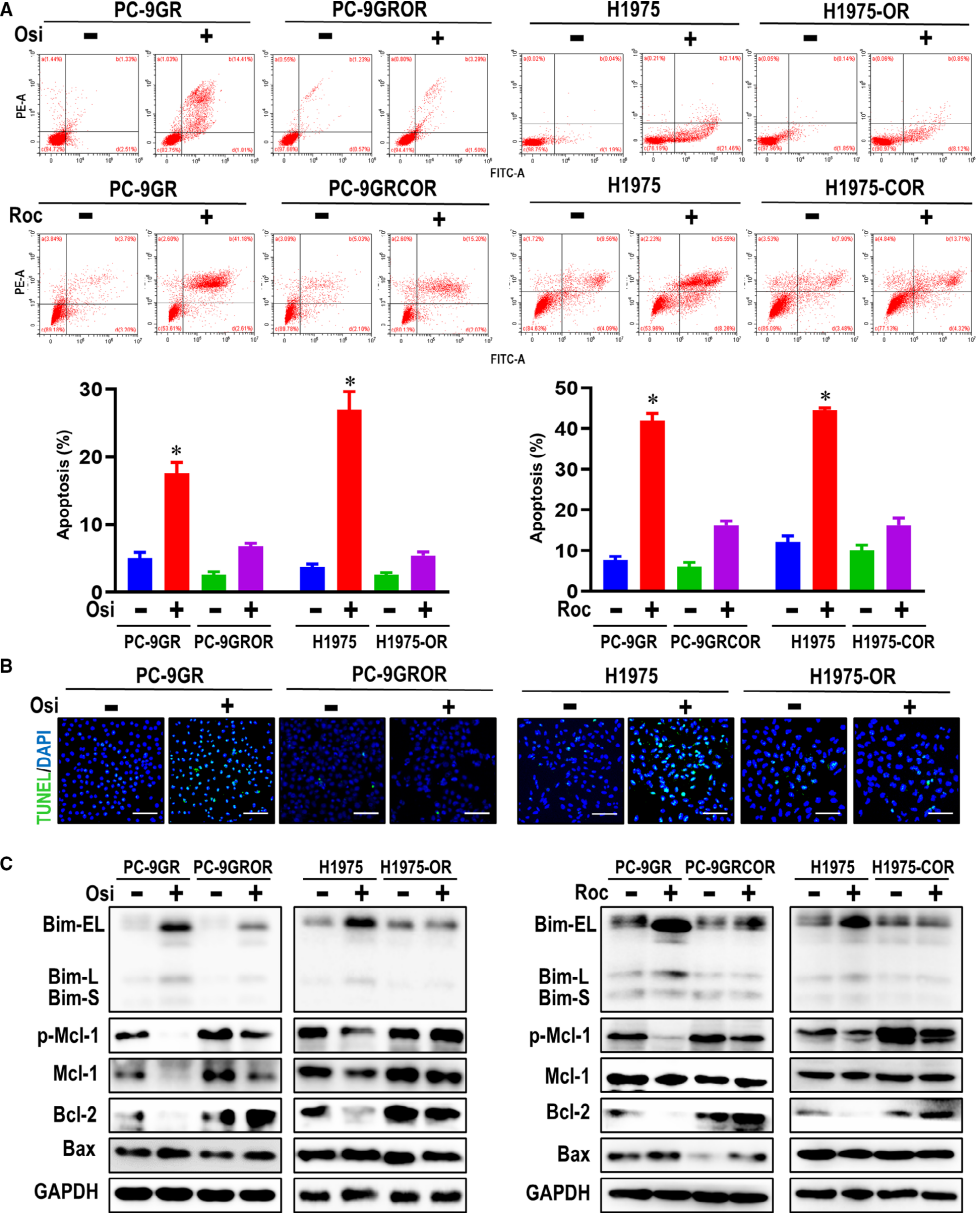
Apoptosis reduction leads to acquired resistance to osimertinib in NSCLC cell lines. (A) The apoptotic parental and resistant cell lines followed with osimertinib and rociletinib treatment or not by flow cytometry. Histogram showed the percentage of apoptotic cells in the indicated groups. *P < 0.05 compared with untreated parental cells. (B) TUNEL staining assessing parental and resistant cell apoptosis after osimertinib treated. (C) The apoptosis‐related proteins were measured by western blot assay. GAPDH was used to confirm equal gel loading.

Reduced apoptosis has been previously demonstrated as an important factor associated with osimertinib resistance (Shi *et al.*, 2017). First, we observed that osimertinib‐resistant cells (PC‐9GROR and H1975‐OR) displayed morphologic features similar to those of parental cells (Fig. S4A). As shown in Fig. 2A, flow cytometry revealed that osimertinib and rociletinib increased the percentage of annexin V‐positive PC‐9GR and H1975 cells. However, treatment with osimertinib or rociletinib produced a dramatic decline in the percentage of annexin V‐positive PC‐9GROR, H1975‐OR, PC‐9GRCOR, and H1975‐COR cells. Similar results in PC‐9GROR and H1975‐OR cells were obtained using the TUNEL assay (Fig. 2B). With osimertinib treatment, there were clearly more apoptotic PC‐9GR and H1975 cells than PC‐9GROR and H1975‐OR cells. Moreover, western blot analysis showed that osimertinib significantly increased expression of the apoptosis‐related protein Bim and decreased that of Mcl‐1 and Bcl‐2 in PC‐9GR and H1975 cells, though osimertinib only slightly increased Bim and decreased Mcl‐1 and Bcl‐2 in PC‐9GROR and H1975‐OR cells. Similar results were observed in the rociletinib‐resistant cell lines PC‐9GRCOR and H1975‐COR. In contrast, BAX was unchanged by osimertinib treatment in all cell lines (Fig. 2C).

Together, these results indicate that apoptosis is reduced in our osimertinib‐resistant NSCLC cell lines and that osimertinib cannot induce changes to apoptosis‐related proteins in these cells. Thus, a reduced ability to induce apoptosis is associated with acquired resistance to osimertinib.

### 3.4 Aspirin induces apoptosis in osimertinib‐resistant NSCLC cell lines

**Fig. 3:**
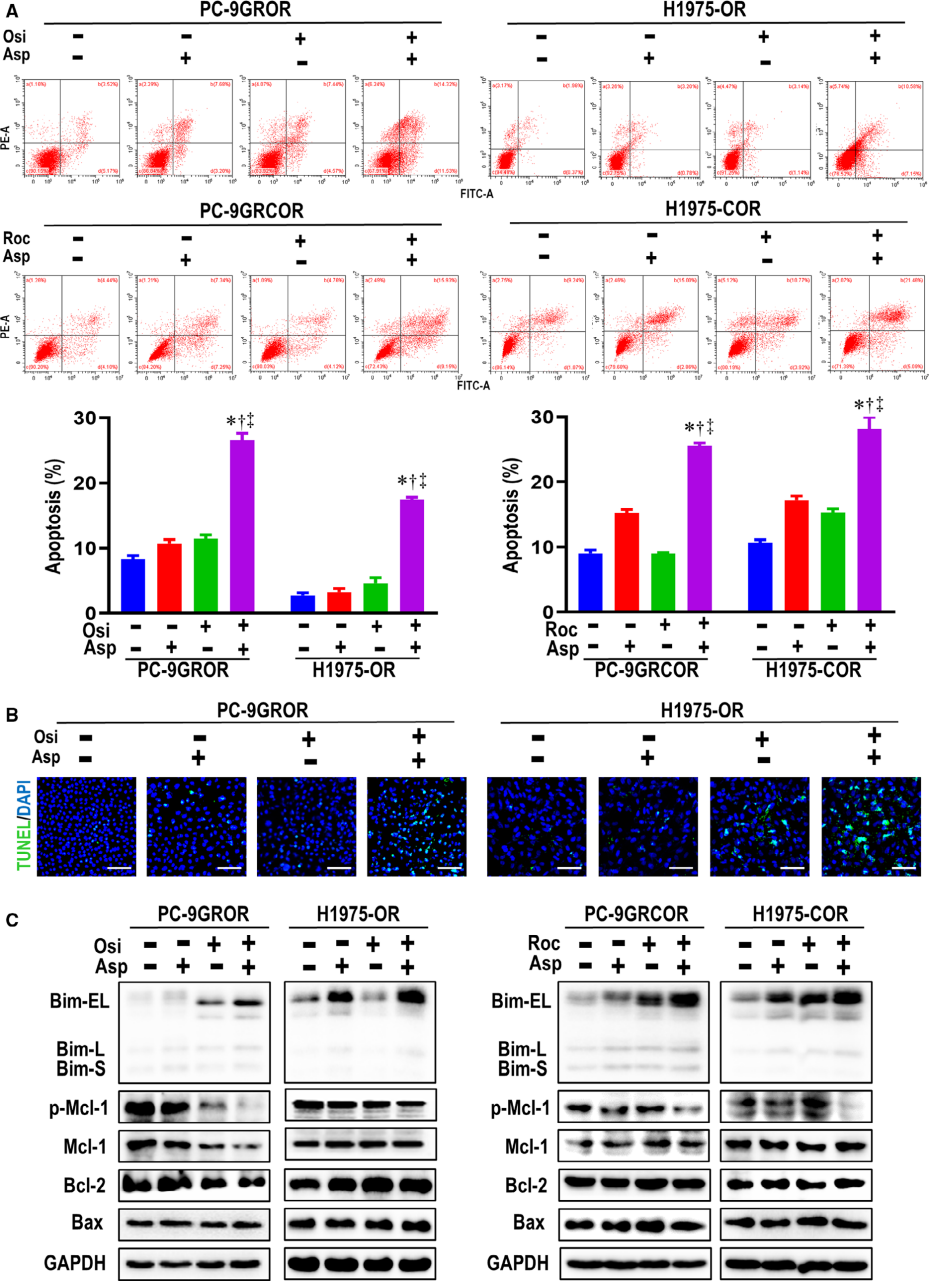
Aspirin induces apoptosis to sensitize osimertinib in NSCLC cell lines. (A) The apoptotic cells after concurrent treated with aspirin and osimertinib/rociletinib were measured with flow cytometry in PC‐9GROR, H1975‐OR, PC‐9GRCOR, and H1975‐COR, respectively. Histogram showed the percentage of apoptotic cells of the indicated groups, respectively.*P < 0.05 compared with control; ^†^ *P* < 0.05 compared with aspirin treatment alone; ^‡^ *P* < 0.01 compared with osimertinib treatment alone. Scale bars: 100 μm. (B) TUNEL staining assessing parental and resistant cell apoptosis after aspirin and osimertinib combined treatment. (C) The critical proteins in Bcl‐2 family including Bim, Mcl‐2, Bcl‐2, and Bax were examined by western blot assay followed with aspirin and osimertinib/rociletinib concurrent treatment in PC‐9GROR, H1975‐OR, PC‐9GRCOR and H1975‐COR cells.

We next examined whether aspirin can increase the antiproliferative effects of osimertinib by inducing apoptosis. We first observed cell morphology and noted no obvious changes after treatment with aspirin or osimertinib alone (Fig. S4B). Flow cytometry showed that although osimertinib or aspirin treatment alone had little effect on apoptosis in either PC‐9GROR or H1975‐OR cells, combining the two drugs significantly enhanced this process in both cell lines, indicating that aspirin further augments the apoptotic effects of osimertinib. Similar phenomenon was also observed in rociletinib‐resistant cell lines when combined treated with aspirin and rociletinib (Fig. 3A). Moreover, TUNEL assay further revealed that the apoptotic PC‐9GROR and H1975‐OR cells were clearly enhanced after exposure to osimertinib and aspirin concurred treatment (Fig. 3B).

According to western blot analysis, aspirin clearly increased Bim expression in both PC‐9GROR and H1975‐OR cells, and the combination of aspirin and osimertinib produced sustained Bim elevation in both cell lines, though Mcl‐1 degradation was obvious only in PC‐9GROR cells (Fig. 3C). Bcl‐2 and BAX levels did not differ significantly following treatment of PC‐9GROR or H1975‐OR cells with aspirin plus osimertinib. However, both Bim elevation and p‐Mcl‐1 suppression were observed in the rociletinib‐resistant cell lines PC‐9GRCOR or H1975‐COR. These findings suggest that Bim elevation may be a common mechanism through which aspirin overcomes osimertinib and rociletinib resistance by blocking proliferation and/or inducing apoptosis in PC‐9GROR and H1975‐OR cells. In gefitinib‐resistant and erlotinib‐resistant cell lines, aspirin also overcome resistance by increasing Bim (Fig. S5A,B).

### 3.5 Bim‐dependent apoptosis is an important mechanism underlying aspirin's ability to resensitize osimertinib in NSCLC cell lines

**Fig. 4:**
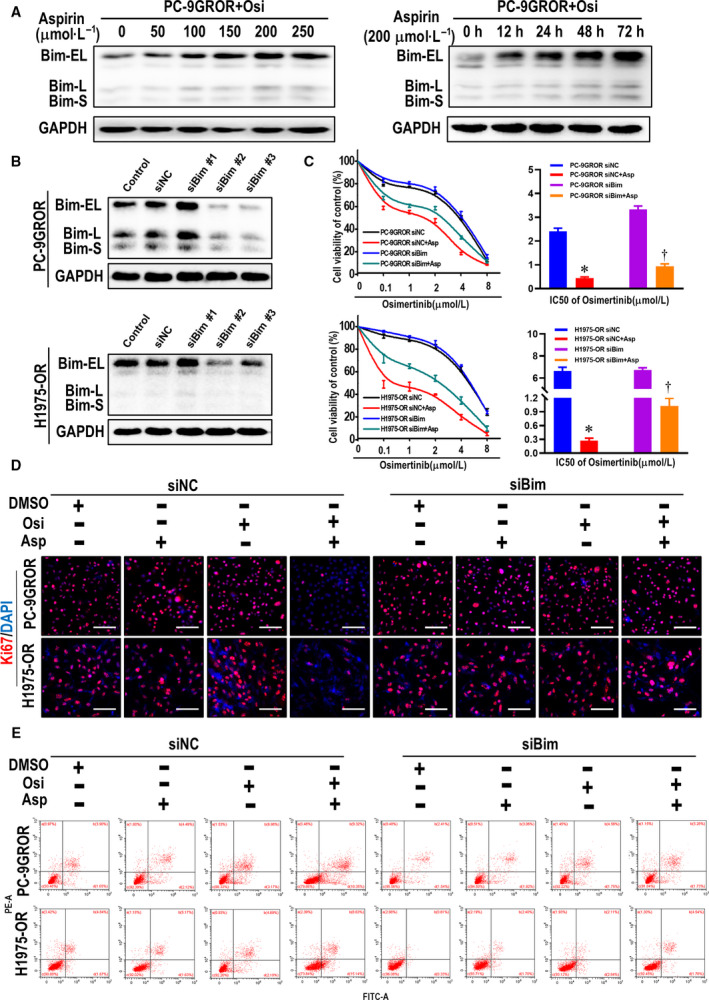
Aspirin induces apoptosis dependent on Bim elevation. (A) PC‐9GROR cells were performed with osimertinib in indicated doses and time points, and Bim proteins were detected by western blot analysis. (B) PC‐9GROR and H1975‐OR cells were transfected with siRNA, and the Bim level was detected by western blot analysis. (C) The cell viability was detected with MTT assay after knocking down Bim expression in PC‐9GROR and H1975‐OR cells. Histogram showing the IC_50_ values for the indicated groups. *P < 0.05 compared with the siNC + osimertinib group; ^†^ *P* < 0.05 compared with the siBim + osimertinib group. (D) The cell proliferation was detected by the Ki67 proliferation assay after knocking down Bim expression in PC‐9GROR and H1975‐OR cells. (E) The apoptotic cells were measured by flow cytometry analysis after knocking down Bim expression in PC‐9GROR and H1975‐OR cells. DAPI, 4′,6‐diamidino‐2‐phenylindole; NC, negative control.

To further confirm the essential role of Bim elevation in mediating aspirin‐induced apoptosis, we first used western blot analyses to detect Bim expression in PC‐9GROR and H1975‐OR cells treated with aspirin for different durations or at different doses. In both cell lines, Bim expression gradually increased in a time‐dependent manner when aspirin was administered for 0 to 72 h and in a dose‐dependent manner when aspirin doses ranged from 0 to 250 μmol·L^−1^ (Fig. 4A and Fig. S6A,B). Subsequently, we used Bim siRNAs to knock down Bim expression in PC‐9GROR and H1975‐OR cells and examined the impact on synergistic growth inhibition by aspirin and osimertinib. As Bim siRNA#2 knocked down Bim expression in both PC‐9GROR and H1975‐OR cells, as confirmed by western blot analyses (Fig. 4B), we used Bim siRNA#2 to treat PC‐9GROR and H1975‐OR cells for 48 h. MTT assays showed that Bim knockdown partially weakened the synergistic effects of aspirin and osimertinib in inhibiting cell proliferation (Fig. 4C). Similarly, the proliferative cells detected by Ki67 assay after knocking down Bim in PC‐9GROR and H1975‐OR cells present no significant difference in combined group than either agent alone (Fig. 4D and Fig. S7A). Whereas when the cells were transfected with Bim siRNA#2, flow cytometry analysis showed the combination of aspirin and osimertinib obviously decreases apoptosis than either agent alone (Fig. 4E and Fig. S7B). Taken together, these results suggest that Bim elevation is an important mechanism underlying the ability of aspirin to resensitize osimertinib by inducing apoptosis in osimertinib‐resistant cells.

### 3.6 Aspirin enhances Bim expression by inhibiting Akt‐FoxO3a signaling in osimertinib‐resistant NSCLC cell lines

**Fig. 5:**
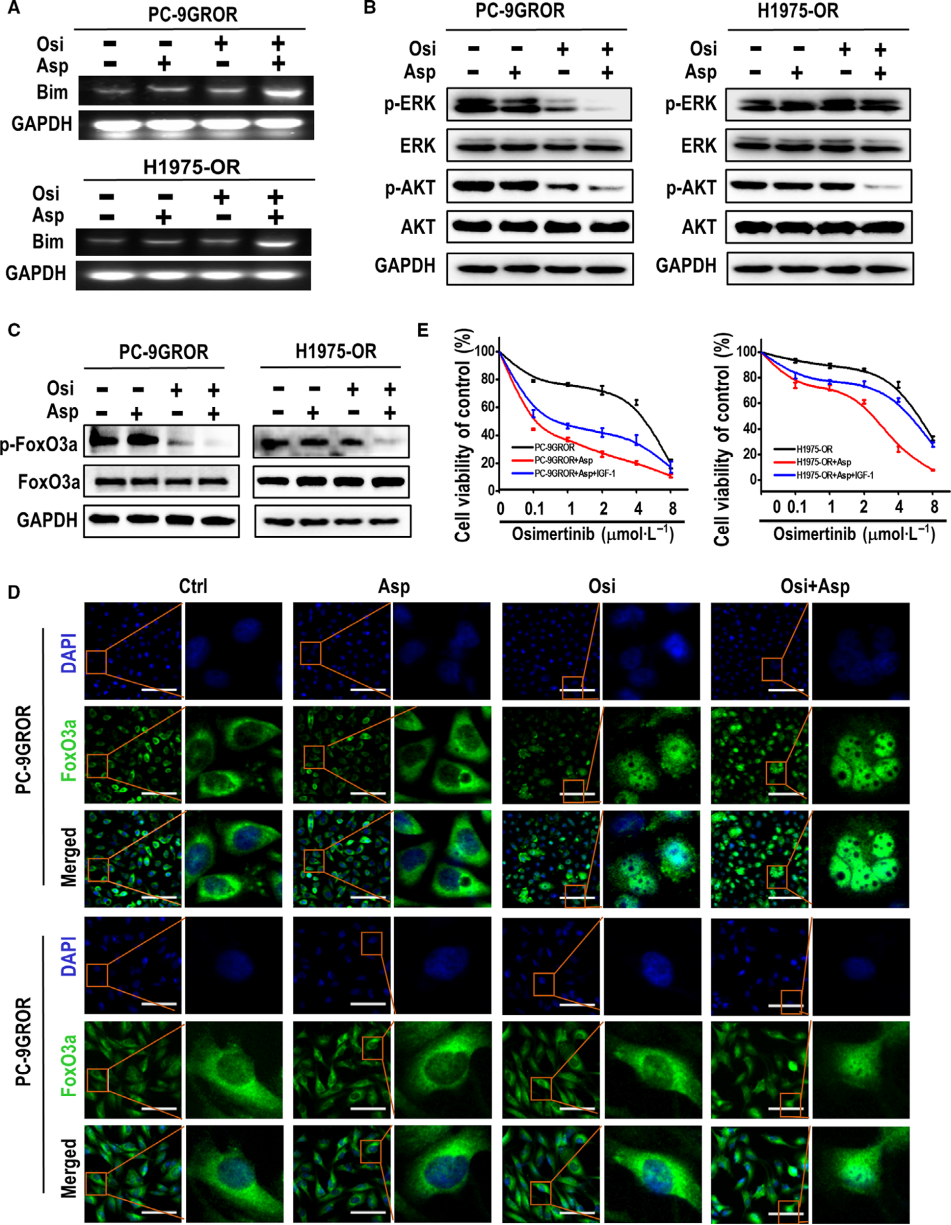
Inhibition of Akt and FoxO3a phosphorylation plays a crucial role in the elevation of Bim expression. (A) Bim transcriptional level was assessed by RT‐PCR in PC‐9GROR and H1975OR cell with the indicated treatment. (B) The Akt and Erk phosphorylation proteins were measured by western blot assay combined treated with aspirin and osimertinib or not in PC‐9GROR and H1975‐OR cells. GAPDH was used to confirm equal gel loading. (C) The FoxO3a phosphorylation was measured by western blot assay combined treated with aspirin and osimertinib or not in PC‐9GROR and H1975‐OR cells. GAPDH was used to confirm equal gel loading. GAPDH was used to confirm equal gel loading. (D) Immunofluorescence staining showed the subcellular localization of FoxO3a in PC‐9GROR and H1975‐OR cells with indicated treatments. (E) Cell viability of PC‐9GROR and H1975‐OR cells treated with osimertinib + aspirin + IGF‐1 (200 ng·mL^−1^) for 48 h was assessed by the MTT assay. All the experiments were measured in triplicate.

As Bim is a critical apoptosis regulatory protein and is strictly mediated by both transcription pattern and post‐translational modification pattern, to better clarify the subtle mechanism of Bim elevation caused by aspirin, we performed RT‐PCR assay in PC‐9GROR and H1975‐OR cells first. According to the RT‐PCR data, Bim mRNA level was significantly enhanced after aspirin concurrent treatment with osimertinib, comparing with aspirin or osimertinib individual treatment, illustrating Bim elevation was modulated by aspirin transcriptionally (Fig. 5A).

Then, we detected the key signaling pathways for Bim transcription regulation. We used western blotting to analyze Akt and Erk phosphorylation in PC‐9GROR and H1975‐OR cells treated for 48 h with aspirin, osimertinib, or both (Petigny‐Lechartier *et al.*, 2017; Wu *et al.*, 2016). Erk phosphorylation was strongly inhibited by the combination of aspirin and osimertinib in PC‐9GROR cells but not in H1975‐OR cells. In contrast, Akt phosphorylation was strongly inhibited by the combination treatment in both cell lines (Fig. 5B). Similar results were observed in PC‐9GR, H1975, PC‐9GRCOR, H1975‐COR, and H1650‐M3 cells (Fig. S8A–C). These results suggest that inhibition of Akt phosphorylation is a common molecular mechanism by which aspirin elevates Bim expression, regardless of the genetic background of the osimertinib‐resistant cells.

Previous studies have reported that Akt/FoxO3a signaling modulates Bim transcription, suggesting that FoxO3a phosphorylation may play a prominent role in regulating Bim protein synthesis (Yue and Sun, 2018). Western blot analysis showed that aspirin decreased FoxO3a phosphorylation in the presence of osimertinib (Fig. 5C). In addition, immunofluorescence staining indicated that FoxO3a is transferred from the cytoplasm to the nucleus during aspirin treatment and that FoxO3a assembly in the nucleus activates Bim protein synthesis (Fig. 5D). Next, to examine the effects of Akt phosphorylation during aspirin, osimertinib, or combination treatment, we administered IGF‐1 (an Akt activator) to PC‐9GROR and H1975‐OR cells and found that FoxO3a phosphorylation was increased but Bim expression decreased following combinatory treatment (Fig. S9). MTT assay results showed that IGF‐1 partially weakened the synergistic effects of aspirin and osimertinib in inhibiting PC‐9GROR and H1975‐OR cell proliferation (Fig. 5E). In summary, the results suggest that aspirin combined with osimertinib modulates Bim expression by inhibiting Akt and FoxO3a phosphorylation.

### 3.7 The combination of aspirin and osimertinib inhibits the growth of osimertinib‐resistant NSCLC xenografts in nude mice

**Fig. 6:**
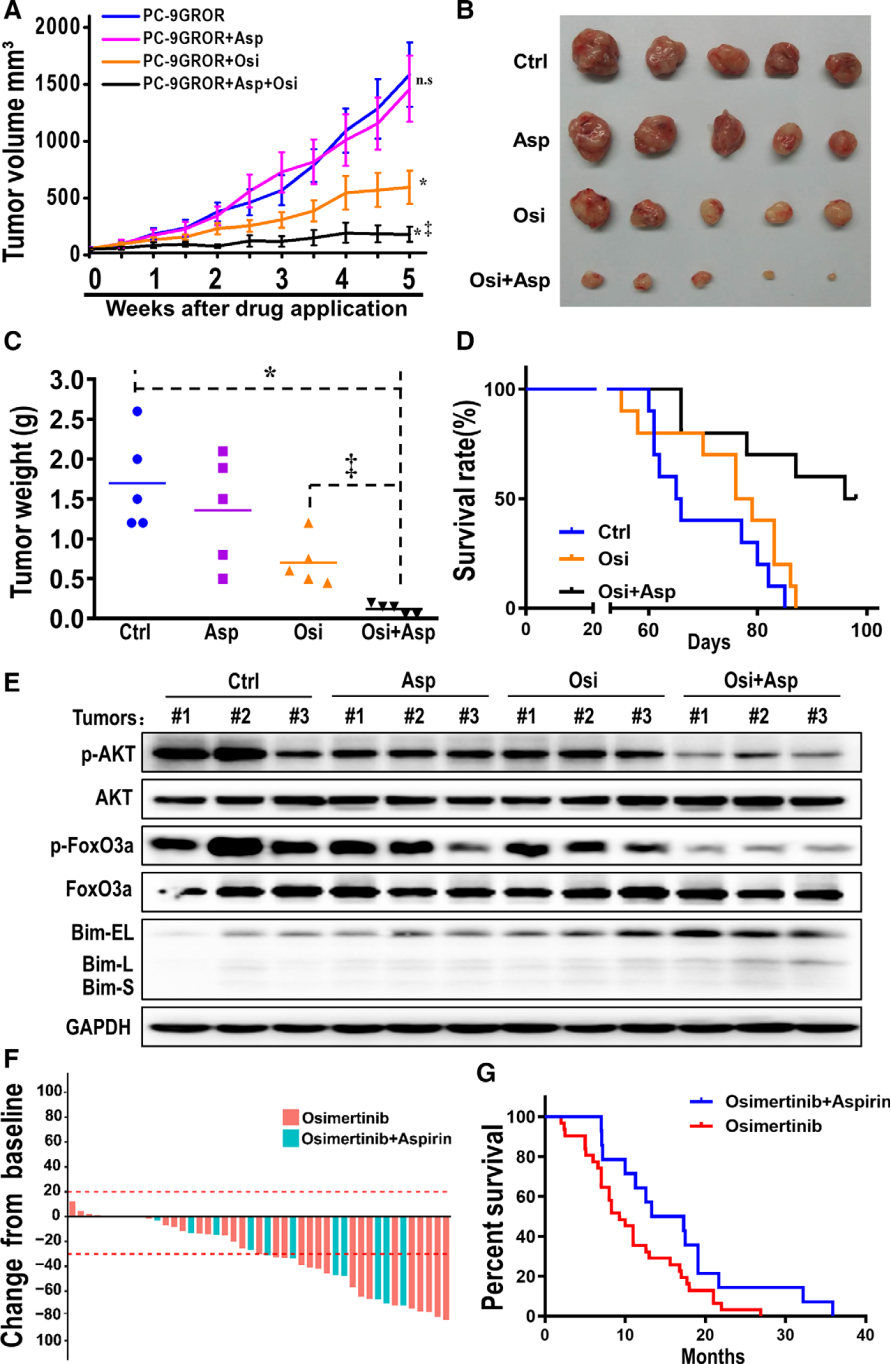
Combination of osimertinib and aspirin effectively inhibited the tumor growth in PC‐9GROR xenografts. (A) Tumor volume (mm^3^) in PC‐9GROR cells with various applications was shown. *P < 0.05 compared with the control group; ^‡^ *P* < 0.01 compared with the osimertinib alone group; n.s: not significant. (B) Macroscopic appearance of tumors after drug application for 5 weeks. (C) Tumor weight (g) in PC‐9GROR cells with various applications was shown. *P < 0.05 compared with the control group; ^‡^ *P* < 0.01 compared with the osimertinib alone group. (D) Kaplan–Meier survival curves were presented for the above three groups. The log‐rank test demonstrated a significant difference between the osimertinib plus aspirin group and the osimertinib alone group. (E) Bim, AKT, and FoxO3a phosphorylation were examined from three tumors randomly selected from each group for western blotting. GAPDH was used as a loading control. (F) Best change from baseline in tumor size (*n* = 45). Dotted lines at 20% and −30% indicate the percent change from baseline and represent progressive disease and PR, respectively, per RECIST v1.1. (G) Kaplan–Meier analysis of NSCLC patients treated with osimertinib plus aspirin and osimertinib alone after the development of first‐generation EGFR‐TKI resistance (PFS analysis).

Based on the above findings, we evaluated whether the combination of aspirin and osimertinib is effective in xenografts established from PC‐9GROR cells. Compared with untreated control mice, mice treated with aspirin or osimertinib alone presented a slightly reduced rate of xenograft tumor growth. Importantly, compared with treatment with either agent alone, treatment with the combination of aspirin and osimertinib significantly reduced tumor growth (Fig. 6A,B). After 5 weeks of drug administration (when the animals were sacrificed), the mean tumor weight in mice bearing PC‐9GROR tumor xenografts treated with aspirin or osimertinib alone was less than that in the control group. Tumor weight was significantly lower following treatment with a combination of aspirin and osimertinib than in all other groups (Fig. 6C). Furthermore, the log‐rank test showed that compared with mice treated with osimertinib alone or those in the control group, mice treated with osimertinib plus aspirin presented significantly prolonged survival (Fig. 6D). No obvious weight loss was observed in any of the mice, including those treated with aspirin plus osimertinib (Fig. S10).

Western blotting was applied to detect protein expression in PC‐9GROR xenografts, and the results showed that phosphorylation of Akt and FoxO3a was strongly inhibited and that Bim expression was significantly elevated in mice treated with the combination compared with mice treated with aspirin or osimertinib alone (Fig. 6E). Collectively, these results indicate that osimertinib plus aspirin significantly slows tumor growth, prolongs survival, and may overcome osimertinib resistance *in vivo* by inhibiting Akt/FoxO3a signaling phosphorylation and increasing Bim expression.

### 3.8 Clinical evidence of combinatorial therapy with osimertinib with aspirin

The retrospective analysis included 45 NSCLC patients with a median age of 59 years (range, 38–84 years). Of these patients, 27 harbored EGFR 19Del and 18 L858R (Table S1). All patients exhibited resistance to a first‐generation EGFR‐TKI (gefitinib or erlotinib) due to the T790M mutation and had received osimertinib treatment. Moreover, 14 patients received osimertinib while taking aspirin due to cardiovascular disease or thrombus, and 31 other patients received osimertinib alone. As shown in Table S1, there were no significant differences in age, sex, or EGFR mutation status between the two groups. Among the 45 patients, 36 (80%) experienced a reduction in measurable tumor size (Fig. 6F). The median PFS was 15.3 and 9.3 months for patients in the osimertinib plus aspirin group and osimertinib group, respectively. The median PFS in the osimertinib plus aspirin group was significantly longer than that in the osimertinib group (*P* = 0.023) (Fig. 6G). We also compared the therapeutic effect of osimertinib on 19Del and L858R mutations, though the difference was not statistically significant in either group (osimertinib plus aspirin group: *P* = 0.487, osimertinib group: *P* = 0.697) (Table S1).

## 4 Discussion

### 4.1 Patient‐reported outcomes from FLAURA

Among patients with previously untreated advanced NSCLC with an EGFR mutation, those who received osimertinib had longer overall survival than those who received gefitinib or erlotinib (38.6 vs 31.8 months) (Ramalingam *et al.*, 2020). Unfortunately, the acquired resistance to osimertinib is inevitable, the molecular mechanism underlying acquired osimertinib resistance was still not fully elucidated (Le *et al.*, 2018), and therapeutic strategies for osimertinib resistance are limited. Previous studies have employed the combination of a first‐generation EGFR‐TKI with osimertinib if the C797S and T790M mutations occurred in cis (Niederst *et al.*, 2015) and the combination of crizotinib with osimertinib if MET amplification was present (Deng *et al.*, 2018). Combination treatment with MEK/BRAF‐V600E inhibitors is another therapeutic strategy to overcome osimertinib resistance (Ho *et al.*, 2017; Shi *et al.*, 2017). These previous studies demonstrate that the use of drug combinations may be a highly effective approach for overcoming osimertinib resistance. In this study, we found that aspirin overcome drug resistance to osimertinib in NSCLC cell lines and that combining aspirin with osimertinib significantly decreased the growth of NSCLC tumor xenografts *in vivo* and prolonged the survival of mice with these tumors. Accordingly, aspirin may be a clinically feasible drug for overcoming osimertinib resistance.

There has been extensive interest in aspirin as a potential anticancer agent since the publication of clinical reports indicating that the NSAID reduces cancer risk and mortality in colon cancer, hepatocellular cancer, and other cancers (Ait Ouakrim *et al.*, 2015; Lin *et al.*, 2018; Simon *et al.*, 2018; Wield *et al.*, 2018). Further evidence suggests that aspirin exerts remarkable antitumor properties in various tumor cells, including lung cancer cells (Khan *et al.*, 2016), and in mouse models (Henry *et al.*, 2017; Liao *et al.*, 2015; Navone *et al.*, 2018). Previous studies have shown that combining aspirin with erlotinib enhanced the effects of erlotinib in killing EGFR‐mutant NSCLC cells (Hu *et al.*, 2018; Song *et al.*, 2018) and that combining aspirin with sorafenib and other drugs significantly suppressed cancer growth and prolonged remission in xenograft models (Amaral *et al.*, 2018; Miao *et al.*, 2018; Xia *et al.*, 2017; Zhang *et al.*, 2017). Our current study likewise demonstrated that concomitant treatment with aspirin can overcome osimertinib resistance. Furthermore, we observed that aspirin can overcome resistance to another third‐generation EGFR‐TKI, rociletinib, and the first‐generation EGFR‐TKIs gefitinib and erlotinib, as reported in another paper (Hu *et al.*, 2018). Together, our results and those of other studies suggest that combining aspirin with osimertinib is a promising new strategy for overcoming osimertinib resistance and improving the survival of patients with NSCLC.

The specific molecular mechanisms by which aspirin overcomes osimertinib resistance have not been fully elucidated. Reduced apoptosis is associated with the development of drug resistance (Chen *et al.*, 2017; Li *et al.*, 2014; Yochum *et al.*, 2018), and in the current study, osimertinib induced apoptosis in EGFR‐mutant NSCLC cell lines but not in osimertinib‐resistant NSCLC cells. These findings are consistent with the results of previous studies (Shi *et al.*, 2017). Furthermore, apoptosis induction has been demonstrated to enhance the effectiveness of TKIs and chemotherapy drugs in inhibiting tumor cell growth (Morgillo *et al.*, 2013; Shukla *et al.*, 2017). Our current results clearly demonstrate that apoptosis induction is a viable strategy for overcoming osimertinib resistance. Bim, a member of the BCL‐2 family of proteins, is a critical modulator of apoptosis (Kang and Reynolds, 2009; Ng *et al.*, 2012). We found that aspirin increased Bim levels in osimertinib‐resistant NSCLC cells; additionally, when Bim expression was knocked down by Bim siRNA, the synergistic effects of aspirin and osimertinib were greatly reduced. Therefore, the ability of aspirin to overcome osimertinib resistance by inducing apoptosis is possibly mediated by increased Bim expression.

Regarding the mechanism by which aspirin modulates Bim expression in osimertinib‐resistant NSCLC cells, previous studies report that Bim induction is a consequence of both post‐translational modification and transcriptional induction, which depend on Akt and the Erk signaling pathway (Petigny‐Lechartier *et al.*, 2017; Wu *et al.*, 2016). A recent study reported that the transcription factor FoxO3a regulates Bim expression in response to many stimuli (Essafi *et al.*, 2005). FoxO3a can also activate the Bim gene directly, disrupt mitochondrial integrity, release cytochrome *c*, promote activation of apoptotic proteins such as caspase‐3, and induce apoptosis in tumor cells (Gilley *et al.*, 2003). In the current study, we observed that dephosphorylated FoxO3a enters the nucleus, where it binds directly to the conserved FoxO‐binding element in the promoter of the Bim gene to induce apoptosis (Vogiatzi *et al.*, 2006). The basal levels of the FoxO3a and Bim proteins (which were already high), as well as Bim mRNA, were increased dramatically after aspirin treatment, and this increase was associated with induction of apoptosis in osimertinib‐resistant NSCLC cells. In addition, previous evidence indicates that phosphorylated Akt or Erk phosphorylates FoxO3a and blocks FoxO3a nuclear entry. We demonstrated that inhibition of Akt phosphorylation can induce apoptosis (and thereby overcome osimertinib resistance) in both PC‐9GROR and H1975‐OR cell lines through dephosphorylated FoxO3a and elevated Bim levels; however, inhibition of Erk phosphorylation by aspirin was just observed in PC‐9GROR cells whereas not in H1975‐OR cells. These results indicate that the aspirin‐mediated increase in Bim expression in PC‐9GROR and H1975‐OR cells was not Erk‐dependent, a result that differs from that reported by Sun *et al*. (Shi *et al.*, 2017). This finding suggests that other common mechanisms such as Akt/FoxO3a signaling may be involved in the effects of aspirin. Therefore, we postulate that aspirin acts by inhibiting phosphorylation of Akt/FoxO3a signaling components and promoting FoxO3a transfer to the nucleus to increase Bim expression and induce tumor cell apoptosis. Moreover, a retrospective analysis was performed in 45 NSCLC patients with acquired resistance to first‐generation EGFR‐TKIs who were administered with osimertinib as the second‐line treatment. The results of this analysis also showed that aspirin can postpone osimertinib resistance. Interestingly, antitumor effects of aspirin were closely correlated with PGE2 reduction (Ma *et al.*, 2017). Therefore, to investigate the probable way aspirin mediating osimertinib resistance, ELISA was performed to observe the PGE2 secretion in osimertinib parental and resistant cells with ELISA kits. Regretfully, there was no significant difference in the level of PGE2 between osimertinib‐resistant and parental cell lines (Fig. S11).

Herein, we describe for the first time the beneficial effects of combined treatment with osimertinib and aspirin in patients with osimertinib resistance and present experimental *in vitro* and *in vivo* data to support and explain this conclusion (Fig. S12). Based on other prior studies, the dosage of aspirin, which was given by oral gavage, used in the *in vivo* study (20 mg·kg^−1^·day^−1^) corresponds to a dose of 60 mg·m^−2^ in humans, which is equivalent to a clinical dose of 100 mg for the average body surface area or approximately one aspirin tablet taken for the purposes of decreasing risk factors for cardiovascular disease in 50‐kg humans. The dose of aspirin used in the *in vitro* study (200 µmol·L^−1^) is in accordance with the IC_25_ in NSCLC cells observed in this study and much lower than that used in most reports (Chen *et al.*, 2018; Hossain *et al.*, 2012; Stark *et al.*, 2007). We have developed three clinical trials, which are registered at ClinicalTrials.gov (NST: 03543683, NST: 03532698, and NST: 04184921), to observe whether aspirin combination with osimertinib could delay the appearance of resistance to osimertinib and the clinical benefits of aspirin combined with osimertinib in treating patients with acquired resistance to first‐generation EGFR‐TKIs or osimertinib. These trials aim to provide solid clinical evidence to further the development of aspirin plus osimertinib as a new combination therapeutic strategy for overcoming acquired osimertinib resistance and prolonging survival in patients with NSCLC.

## 5 Conclusions

In summary, this study demonstrates that the combination of aspirin and osimertinib can overcome osimertinib resistance *in vitro* and *in vivo*. Aspirin, an ‘old drug’, has been used as an analgesic anti‐inflammatory medication for more than a century and is known for its safety, reliability, and low cost. We have now discovered that aspirin plays a crucial role in overcoming osimertinib resistance by inhibiting phosphorylation of Akt/FoxO3a signaling components and up‐regulating Bim protein synthesis in osimertinib‐resistant EGFR‐mutated NSCLC cells. Modulating Bim transcription may not be the only mechanism by which aspirin overcomes osimertinib resistance in EGFR‐mutated NSCLC, and in‐depth clinical studies will continue to identify other potential markers linked to aspirin's beneficial effects. Based on our findings, we suggest that combining aspirin with osimertinib may be an effective and economical therapeutic strategy for overcoming acquired osimertinib resistance.

## Conflict of interest

The authors declare no conflict of interest.

## Author contributions

YH conceived and designed the project. RH directly supervised the study, designed experiments, and wrote the paper. RH, SH, C Lu, and CZ acquired the data. C Lin, CH, and YW participated in discussion of the project. YH and LL directly supervised the study.

## Supporting information


**Fig. S1.** The effects of different doses of aspirin on various cell lines.
**Fig. S2.** The chest CT scan presented another patient's pulmonary nodule progression.
**Fig. S3.** The effect of aspirin combined with different EGFR‐TKIs in different cell lines.
**Fig. S4.** The morphological features in osimertinib sensitive‐ and resistant‐ cells with osimertinib combined with or without aspirin treatment.
**Fig. S5.** Aspirin overcome resistance by increasing Bim level in gefitinib and erlotinib resistant cell respectively.
**Fig. S6.** H1975‐OR cells were performed with osimertinib concurrend with indicated doses.
**Fig. S7.** Histograms of Ki67 cell proliferation alterations and of flow cytometry cell apoptosis alterations.
**Fig. S8.** Western blotting to analyze Akt and Erk phosphorylation in PC‐9GR, H1975, H1650‐M3, PC‐9GRCOR and H1975COR cell lines.
**Fig. S9.** The expression of AKT, p‐AKT, FoxO3a, p‐FoxO3a, Bim were measured by western blot assay in PC‐9GROR and H1975‐OR cell lines.
**Fig. S10.** The nude mice body weight.
**Fig. S11.** PGE2 secretion in osimertinib parental‐ and resistant‐ cells with ELISA assay.
**Fig. S12.** The schematic diagram for the mechanism that aspirin overcomes osimertinib resistance.

**Fig. S13.** The expression of AKT, p‐AKT, FoxO3a, p‐FoxO3a, ERK, p‐ERK were measured by western blot assay in osimertinib parental and resistant cell respectively.
**Fig. S14.** (A) The role of aspirin in resensitivity to osimertinib in osimertinib sensitive PC‐9GR cells. (B) Histogram shows IC50 of osimertinib in the indicated groups.

**Table S1.** The patient characteristics of 45 patients presenting with NSCLC.

## Data Availability

No data deposited in public database or repository.
