# Supplementary material for: Aspirin sensitizes osimertinib‐resistant NSCLC cells in vitro and in vivo via Bim‐dependent apoptosis induction
Source: Mol Oncol. 2020 May 5;14(6):1152–69. doi: 10.1002/1878-0261.12682 (PMC7266273; doi:10.1002/1878-0261.12682)
Supplement: Supplementary file 1 — Fig. S1. The effects of different doses of aspirin on various cell lines. Fig. S2. The chest CT scan presented another patient's pulmonary nodule progression. Fig. S3. The effect of aspirin combined with different EGFR‐TKIs in different cell lines. Fig. S4. The morphological features in osimertinib sensitive‐ and resistant‐ cells with osimertinib combined with or without aspirin treatment. Fig. S5. Aspirin overcome resistance by increasing Bim level in gefitinib and erlotinib resistant cell respectively. Fig. S6. H1975‐OR cells were performed with osimertinib concurrend with indicated doses. Fig. S7. Histograms of Ki67 cell proliferation alterations and of flow cytometry cell apoptosis alterations. Fig. S8. Western blotting to analyze Akt and Erk phosphorylation in PC‐9GR, H1975, H1650‐M3, PC‐9GRCOR and H1975COR cell lines. Fig. S9. The expression of AKT, p‐AKT, FoxO3a, p‐FoxO3a, Bim were measured by western blot assay in PC‐9GROR and H1975‐OR cell lines. Fig. S10. The nude mice body weight. Fig. S11. PGE2 secretion in osimertinib parental‐ and resistant‐ cells with ELISA assay. Fig. S12. The schematic diagram for the mechanism that aspirin overcomes osimertinib resistance. [file MOL2-14-1152-s001.pdf]

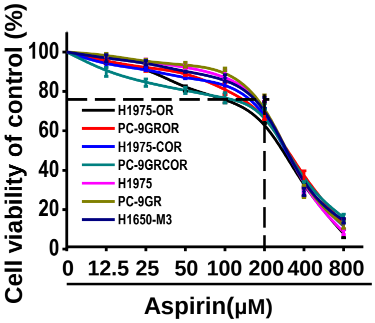

Supplementary Figure 1. The effects of different doses of aspirin on various cell lines. Cell viabilities were examined at 48 h after aspirin treatment in osimertinib sensitive- and resistant- cell lines. Assays were repeated in triplicate. Mean ± SD.

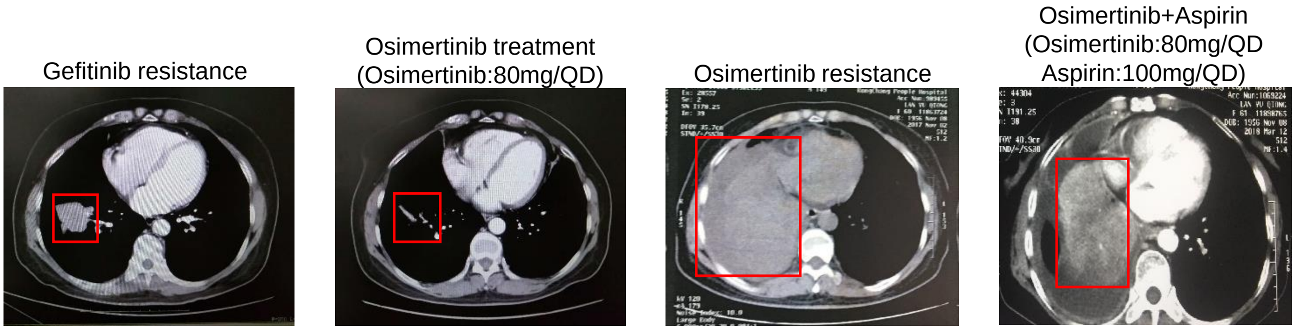

Supplementary Figure 2. The chest CT scan presented another patient's pulmonary nodule progression after osimertinib treatment and combined with aspirin treatment respectively.

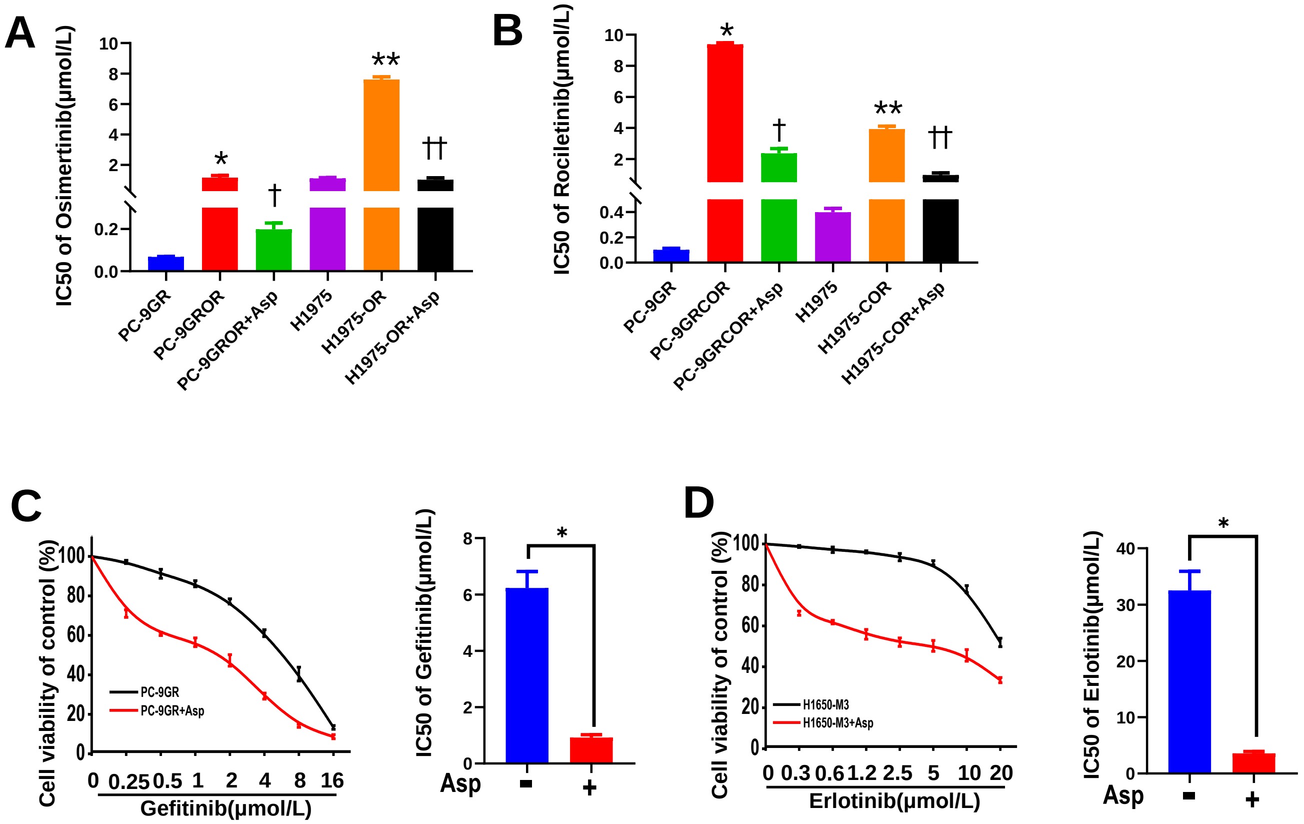

Supplementary Figure 3. The effect of aspirin combined with different EGFR-TKIs in different cell lines. (A, B)Histogram shows IC50 of osimertinib and rociletinib in the indicated groups respectively. (C) Cell viability was analyzed by MTT assay in PC-9GR cells treated with gefitinib alone and combined with aspirin treatment for 48 h. Histogram shows IC50 of gefitinib in the indicated groups. (D) Cell viability was analyzed by MTT assay in H1650-M3 cells treated with erlotinib alone and combined with aspirin treatment for 48 h. Histogram shows IC50 of erlotinib in the indicated groups. All the assays were repeated in triplicate. Mean ± SD, *p < 0.05, †p < 0.05; **p <0.01, ††p < 0.01. Asp: aspirin.

A

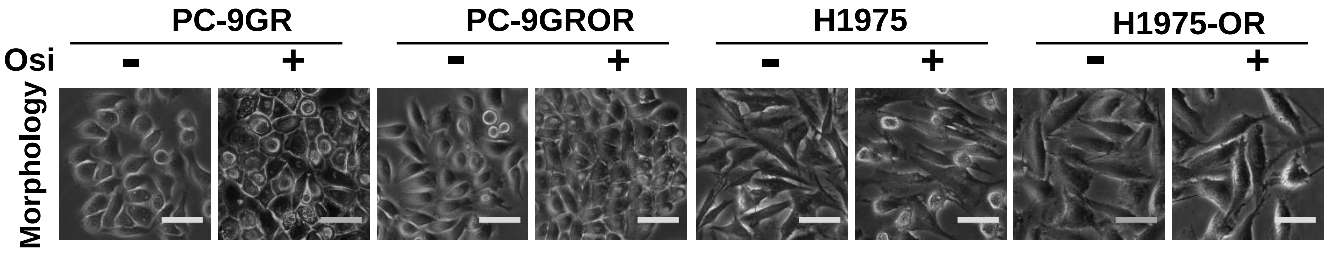

B

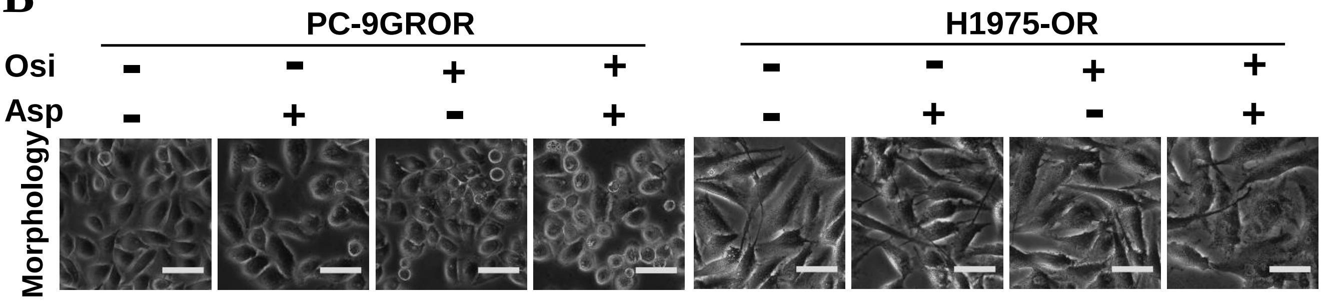

Supplementary Figure 4. The morphological features in osimertinib sensitive- and resistant- cells with osimertinib combined with or without aspirin treatment. (A) morphological characters of parental and resistant cells in the presence of osimertinib shown by phase-contrast images. (B) The morphological features of osimertinib resistant cells lines with different treatments shown by phase-contrast images. Scale bars: 50μm. Asp: aspirin; Osi: osimertinib.

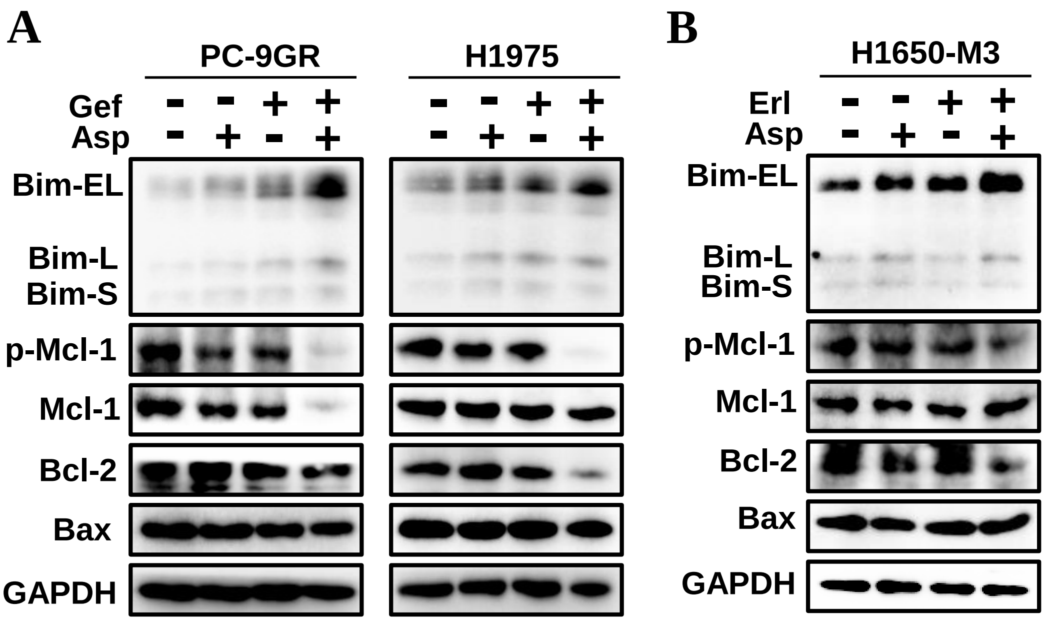

Supplementary Figure 5. Aspirin overcome resistance by increasing Bim level in gefitinib and erlotinib resistant cell respectively. (A) The expression of Bim, p-Mcl-1 and Bcl-2 were measured by western blot assay in PC-9GR and H1975 cell lines by gefitinib concurred with aspirin treatment or not for 48 h. (B) The expression of Bim, p-Mcl-1 and Bcl-2 were measured by western blot assay in H1650-M3 cell by erlotinib concurred with aspirin treatment or not for 48 h. All experiments were repeated in triplicate and shown as mean ± SD. Asp: aspirin; Gef: gefitinib; Erl: erlotinib.

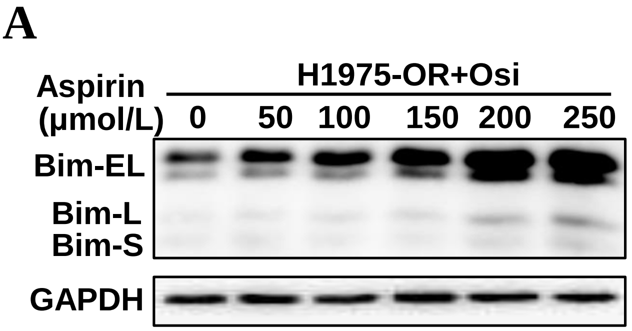

Supplementary Figure 6. H1975-OR cells were performed with osimertinib concurrend with indicated doses (A) and indicated time points (B) of aspirin treatment. Bim protein levels were detected by Western blot assay. All experiments were repeated in triplicate and shown as mean ± SD. Osi: osimertinib.

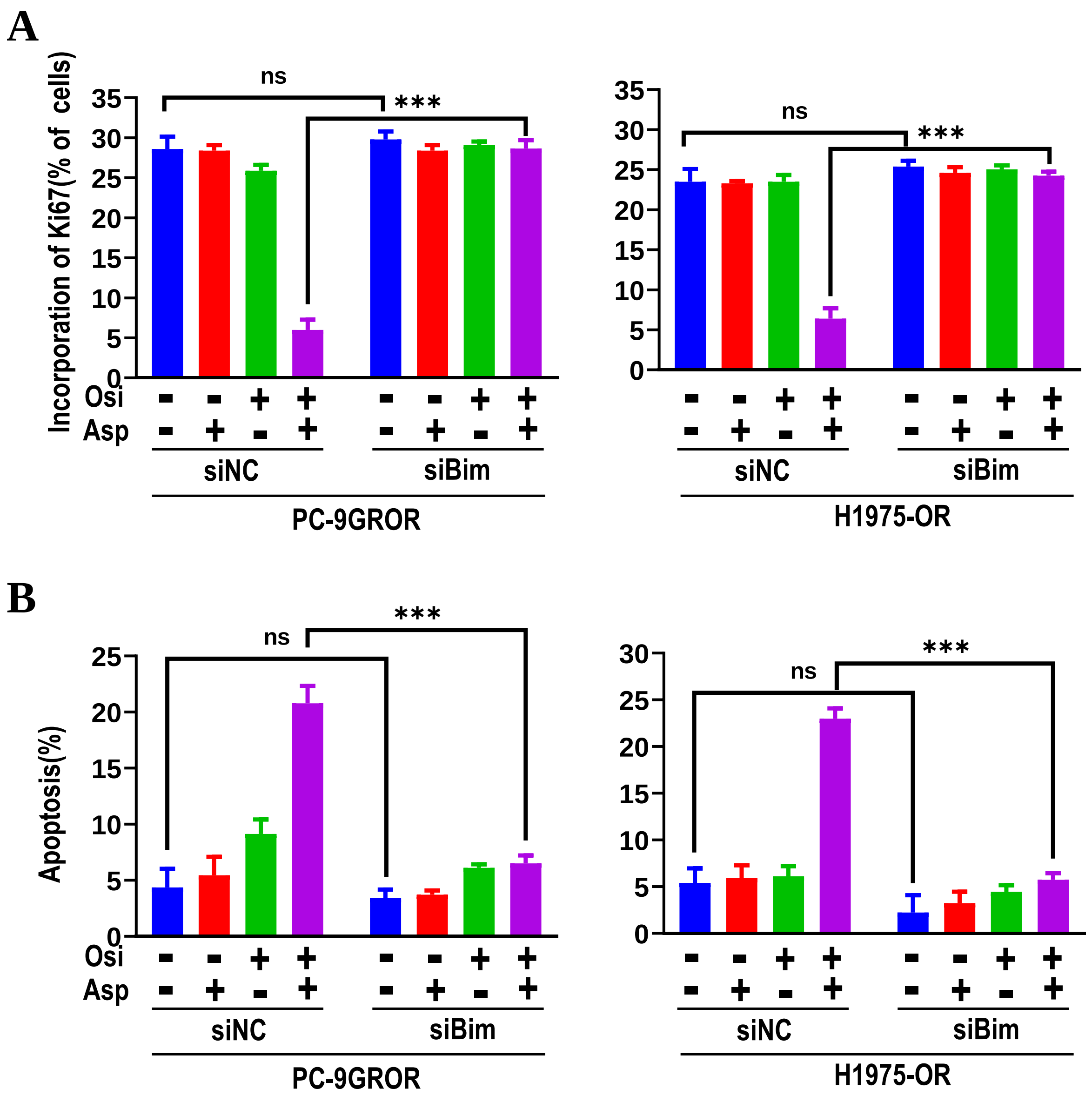

Supplementary Figure 7. (A) Histograms of Ki67 cell proliferation alterations were shown after knockdown of Bim in the indicated groups of PC-9GROR and H1975-OR cells. (B) Histograms of flow cytometry cell apoptosis alterations were shown after knockdown of Bim in the indicated groups of PC-9GROR and H1975-OR cells. Experiments were repeated in triplicate and shown as mean ± SD, ***$p < 0.001$. ns: no significance.

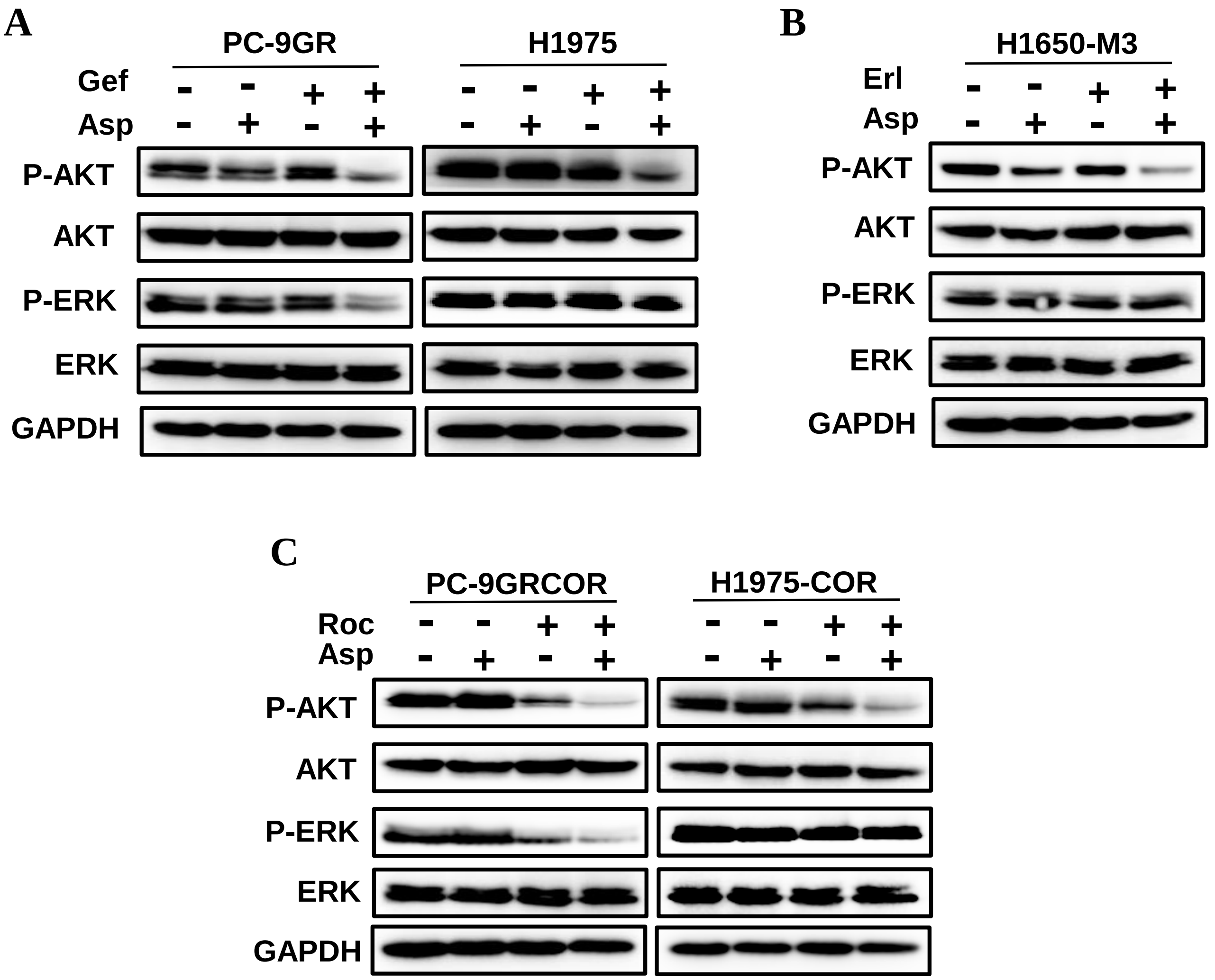

Supplementary Figure 8. (A) The expression of AKT, p-AKT, ERK, p-ERK were measured by western blot assay in PC-9GR and H1975 cell lines by gefitinib (IC_{25}^{48h}) concurred with aspirin treatment or not for 48 h. (B) The expression of AKT, p-AKT, ERK, p-ERK were measured by western blot assay in H1650-M3 cell by erlotinib (IC_{25}^{48h}) concurred with aspirin treatment or not for 48 h. (C) The expression of AKT, p-AKT, ERK, p-ERK were measured by western blot assay in PC-9GRCOR and H1975-COR cells by rociletinib (IC_{25}^{48h}) concurred with aspirin treatment or not for 48 h. All experiments were repeated in triplicate. Asp: aspirin; Gef: gefitinib; Erl: erlotinib; Roc: rociletinib.

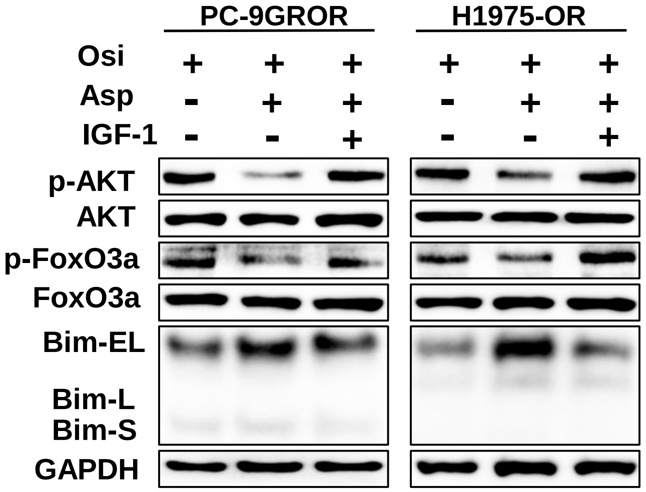

Supplementary Figure 9. The expression of AKT, p-AKT, FoxO3a, p-FoxO3a, Bim were measured by western blot assay in PC-9GROR and H1975-OR cell lines exposed to osimertinib (IC_{25}) and IGF-1 (200ng/mL) with or without aspirin (200μmol/L) treatment for 48 h. Experiments were repeated in triplicate. Asp: aspirin; Osi: osimertinib.

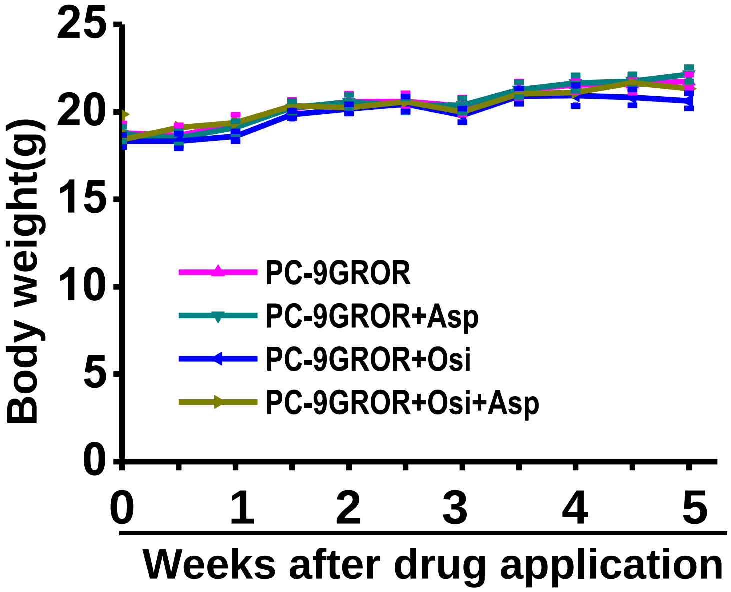

Supplementary Figure 10. The nude mice body weight were presented after indicated treatment and the data was shown as mean ± SEM (n=5). Asp: aspirin; Osi: osimertinib.

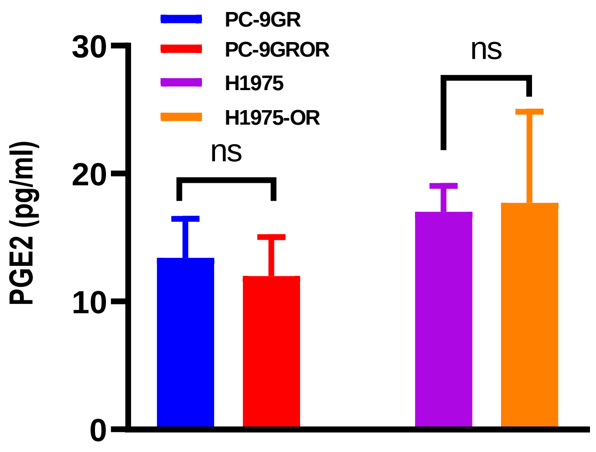

Supplementary Figure 11. PGE2 secretion in osimertinib parental- and resistant-cells with ELISA assay. Experiments were repeated in triplicate. ns: no significance

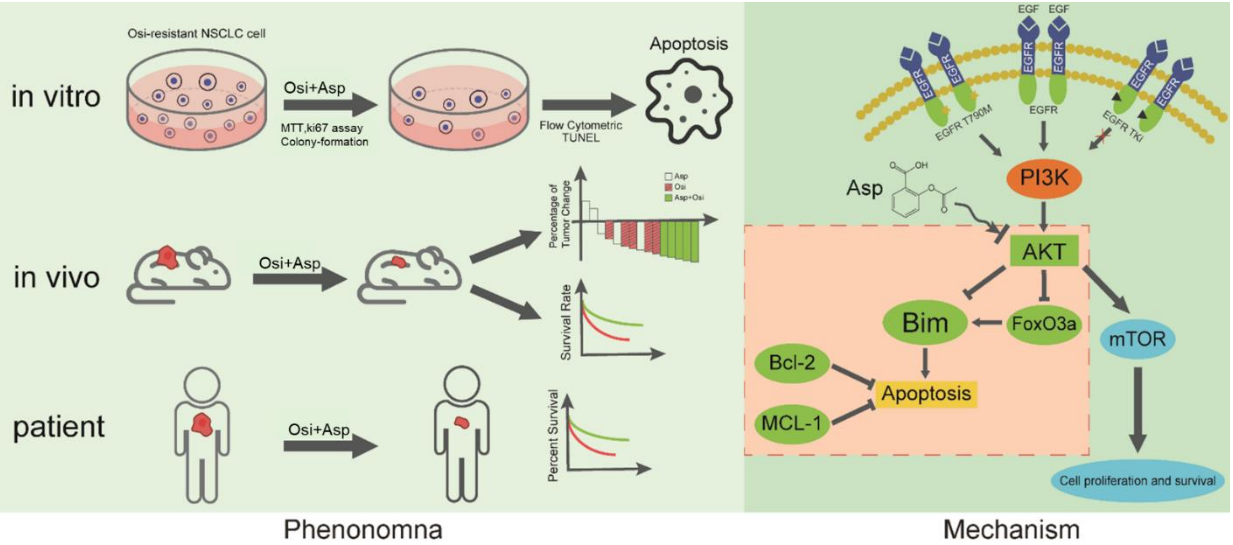

Supplementary Figure 12. The schematic diagram for the mechanism that aspirin overcomes osimertinib resistance via Bim-dependent apoptosis induction in osimertinib resistant NSCLC cells.
