# Supplementary material for: Aspirin sensitizes osimertinib‐resistant NSCLC cells in vitro and in vivo via Bim‐dependent apoptosis induction
Source: Mol Oncol. 2020 May 5;14(6):1152–69. doi: 10.1002/1878-0261.12682 (PMC7266273; doi:10.1002/1878-0261.12682)
Supplement: Supplementary file 2 — Fig. S13. The expression of AKT, p‐AKT, FoxO3a, p‐FoxO3a, ERK, p‐ERK were measured by western blot assay in osimertinib parental and resistant cell respectively. Fig. S14. (A) The role of aspirin in resensitivity to osimertinib in osimertinib sensitive PC‐9GR cells. (B) Histogram shows IC50 of osimertinib in the indicated groups. [file MOL2-14-1152-s002.pdf]

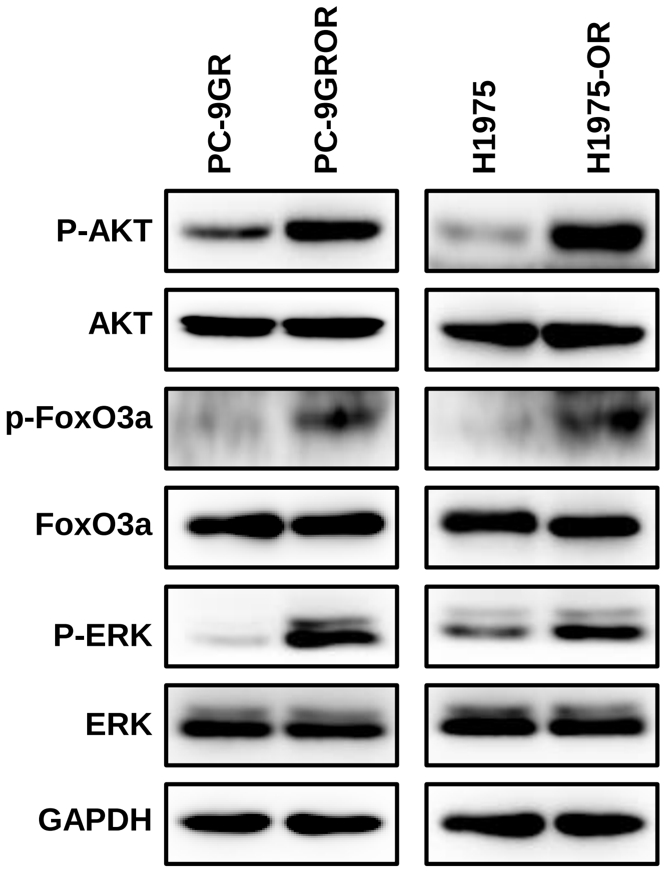

Supplementary Figure 13. The expression of AKT, p-AKT, FoxO3a, p- FoxO3a, ERK, p-ERK were measured by western blot assay in osimertinib parental and resistant cell respectively.

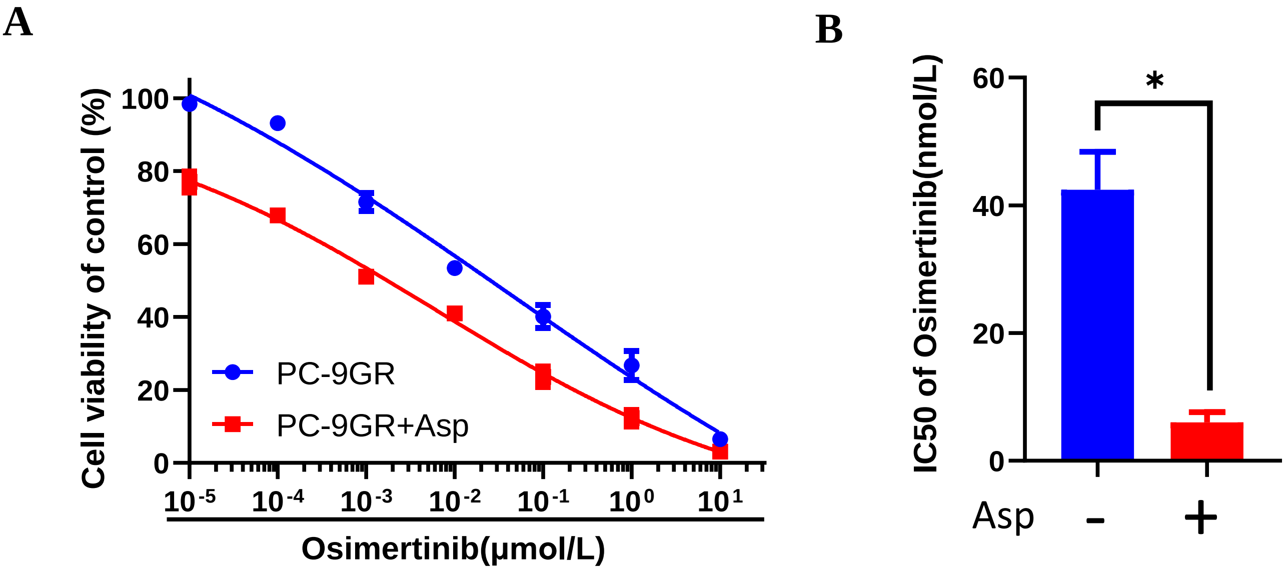

Supplementary Figure 14. (A) The role of aspirin in resensitivity to osimertinib in osimertinib sensitive PC-9GR cells. **(B)** Histogram shows IC_{50} of osimertinib in the indicated groups. Experiments were repeated in triplicate.
