# Supplementary material for: Aspirin sensitizes osimertinib‐resistant NSCLC cells in vitro and in vivo via Bim‐dependent apoptosis induction
Source: Mol Oncol. 2020 May 5;14(6):1152–69. doi: 10.1002/1878-0261.12682 (PMC7266273; doi:10.1002/1878-0261.12682)
Supplement: Supplementary file 3 — Table S1. The patient characteristics of 45 patients presenting with NSCLC. [file MOL2-14-1152-s003.pdf]

Table S1. Patient Characteristics

Characteristics	No. of Patients (%)			
	Total (N=45)	Osimertinib+Aspirin (n=14)	Osimertinib (n=31)	*P*
Age, y				
Median (range)	59 (38-84)	58 (38-84)	60 (43-74)	0.995
<65	32 (71.1)	11 (78.6)	21 (67.7)	
≥65	13 (28.9)	3 (21.4)	10 (32.3)	
Gender				0.355
Male	21 (46.7)	8 (57.1)	13 (41.9)	
Female	24 (53.3)	6 (42.9)	18 (58.1)	
EGFR mutation				0.120
19del	27 (60.0)	6 (42.9)	21 (67.7)	
L858R	18 (40.0)	8 (57.1)	10 (32.3)	
PFS				
Median (range)	11.0 (2.0-35.9)	15.3 (7.0-35.9)	9.3 (2.0-26.9)	0.023
